# *Ab initio* GO-based mining for non-tandem-duplicated functional clusters in three model plant diploid genomes

**DOI:** 10.1371/journal.pone.0234782

**Published:** 2020-06-19

**Authors:** Paolo Bagnaresi, Luigi Cattivelli

**Affiliations:** CREA Research Centre for Genomics and Bioinformatics, Fiorenzuola d’Arda, Italy; Instituto Agricultura Sostenible, SPAIN

## Abstract

A functional Non-Tandem Duplicated Cluster (FNTDC) is a group of non-tandem-duplicated genes that are located closer than expected by mere chance and have a role in the same biological function. The identification of secondary-compounds–related FNTDC has gained increased interest in recent years, but little *ab-initio* attempts aiming to the identification of FNTDCs covering all biological functions, including primary metabolism compounds, have been carried out. We report an extensive FNTDC dataset accompanied by a detailed assessment on parameters used for genome scanning and their impact on FNTDC detection. We propose 70% identity and 70% alignment coverage as intermediate settings to exclude tandem duplicated genes and a dynamic scanning window of 24 genes. These settings were applied to rice, arabidopsis and grapevine genomes to call for FNTDCs. Besides the best-known secondary metabolism clusters, we identified many FNTDCs associated to primary metabolism ranging from macromolecules synthesis/editing, TOR signalling, ubiquitination, proton and electron transfer complexes. Using the intermediate FNTDC setting parameters (at *P-value* 1e^-6^), 130, 70 and 140 candidate FNTDCs were called in rice, arabidopsis and grapevine, respectively, and 20 to 30% of GO tags associated to called FNTDC were common among the 3 genomes. The datasets developed along with this work provide a rich framework for pinpointing candidate FNTDCs reflecting all GO-BP tags covering both primary and secondary metabolism with large macromolecular complexes/metabolons as the most represented FNTDCs. Noteworthy, several FNTDCs are tagged with GOs referring to organelle-targeted multi-enzyme complex, a finding that suggest the migration of endosymbiont gene chunks towards nuclei could be at the basis of these class of candidate FNTDCs. Most FNTDC appear to have evolved prior of genome duplication events. More than one-third of genes interspersed/adjacent to called FNTDCs lacked any functional annotation; however, their co-localization may provide hints towards a candidate biological role.

## Introduction

Functional Non-Tandem Duplicated Clusters (FNTDCs) are groups of non-tandem-duplicated genes having a role in the same biological function located on the genome closer than expected by mere chance [1–5]. A stringent but arbitrary definition of a perfect functional cluster has been proposed, consisting in the presence in the cluster of at least three genes with a different role applied to the same biological function, while a more relaxed description indicates at least two co-localized non-homologous functionally related genes [3].

Recently, we reported the *ab–initio*, unbiased genome scan for FNTDC applied to the tetraploid genome of durum wheat, a work resulting in the identification of 197 candidate FNTDCs covering a wide range of primary and secondary metabolic functions [6]. Eighty-four of them were found to have an homeologous counterpart in the two genomes. We now report the FNTDC scanning for rice, grape and arabidopsis, three phylogenetically distinct plant species (grasses, trees, cruciferous) all endowed with very high-quality genomes and plenty of sequence information, probably the best examples in their respective categories.

A subset of FNTDC, namely the Biosynthetic Gene Clusters (BGCs), are of especial interest since they contain genes with a role in the biosynthetic pathways leading to secondary metabolites with a wide range of applications in diverse fields. This interest has prompted the development of comprehensive bioinformatic tools (e.g. *plantismash* [5, 7], *Phytoclust* [8]) for thorough analyses based on libraries of profile Hidden Markov Models (pHMMs) of enzyme gene families involved in plant biosynthetic pathways. Admittedly, however, these tools are conceived to target biosynthetic gene cluster for secondary metabolism and thus fail to detect the entirety of FNTDC in a broad sense, i.e. clusters of non-tandem genes which share any biological function as specified by the GO biological process tags. Noteworthy, this limitation implies failure to detect potential primary metabolism clusters e.g. the clusters related to nucleic acid, vitamins, carbohydrates, proteins and lipid biosynthesis and maintenance or cofactor biosynthesis. Hints for several primary metabolic clusters have been obtained in further searches which were, again, solely based on enzyme classified on predicted catalytic functions, despite covering larger enzyme classes [1 and references therein], [9].

It is well known that at least part of the genes belonging to a FNTDC can be co-expressed and co-expression data have been leveraged in several works for the identification of the clusters [10]. Indeed, it has been noted that co-expression of adjacent genes, even functionally unrelated, occurs much more frequently than expected by chance [11], a finding associated to chromatin decondensation, as indicated by chromatin signatures of histone 3 lysine trimethylation (H3K27me3) (repression) and histone 2 variant H2A.Z (activation). Detection of such chromatin signatures themselves could pose the basis for genome scanning to pinpoint further functional clusters [12].

Co-expression data for mining FNTDCs has been implemented in the context of the *plantismash* software dedicated to BGC detection [5] helping to identify pathways split over different clusters and genomic locations. In this analysis, however, the focus is the pathway itself and the co-localization requirement for the cluster is set aside. Co-expression of the genes within a candidate cluster reinforces its cluster nature, although it has not been observed for al BGCs, as the conditions prompting (co-)expression may be complex, not yet tested or undetectable in expression databases [5, 7]. For instance, genes involved in multifurcated pathways might not be co-expressed although part of a common FNTDC [5, 7, 13, 14]. All the above observations lead to assessment of co-expression as a complementary (and not compulsory) aspect with optional implementation in some tools as *Phytoclust* [8].

Among the evolutionary forces underpinning the formation and maintenance of FNTDCs, coordinated gene expression, coinheritance and avoidance of accumulation of toxic intermediate compounds are thought to play a major role [2, 15]. The genes belonging to FNTDCs have frequently a role in the secondary metabolism or form protein-protein interaction networks [16] and co-localization could sustain easily tuneable co-expression. The mechanisms governing the accumulation of functionally related genes in close proximity are largely unknown, nevertheless segmental duplication followed by neo-functionalization is typical evolutionary process that allows duplicated genes to gain similar but non-identical functions [1].

Despite the raising interest in functional clusters, both for understanding basic driving forces for genome plasticity and technological application (i.e. the BGC), most recent FNTDC detection attempts do not rely on *ab-initio* un-supervised and systematic scanning of whole genomes covering all biological functions (any gene with an associated biological GO BP process). In fact, such attempts identify enzymes based on phMM profile libraries [5] of specific enzyme classes. In principle, *ab initio* approaches would have a tremendous impact as very large sets of biological functions can be tested scanning one or more genomes with the potential to uncover a far larger set of FNTDCs than currently known. The advances in genomic sequencing and GO-based functional annotations can provide the basis for wide functional and cross-species annotations rather than target only specific enzyme classes. However, an *ab initio* approach requires many computationally intensive GO hypergeometric tests with differently sized sliding windows covering various subsets of gene numbers and frame positioning and testing several combinations in order to fine tune FNTDC size and positioning. Tandem duplicated genes must be identified and reduced to one representative member even if no clear-cut boundaries are defined since genuine tandem duplicates can present large indels while, on the other hand, few amino acid changes can pose the basis for gaining a novel function. Annotation procedures and quality as available in genome data repositories vary deeply and such poor consistency in annotations may hamper comparative analyses. Finally, very large subsets of genes in genomes do not have associated function, precluding any classification based on GO which is the obvious prerequisite for functional cluster scanning. Noteworthy, in Arabidopsis, the biological role of one-third of the proteins was reported as unknown [17] and in-depth analyses stated that the functions of at least 60% of predicted Arabidopsis enzymes and transporters were considered poorly clear or totally unknown [18]. The same authors concluded that “only about one-half of all Arabidopsis genes are characterized to any extent” [18].

In this work we report FNTDC datasets and document the implementation of a state-of-the-art pipeline that tackles the limitations described above. The pipeline starts from updated, standardized, consistent and fine-grained GO BP annotations and implements exclusion of all but one member of tandem duplicated genes according to several levels of stringency. Each stringency level produces datasets ranging from ones highly enriched in genuine FNTDCs to datasets including tandem duplicates with known function. A heuristic procedure explores processively for GO enrichments many window sizes and positioning to fine tune the coordinates of the candidate FNTDCs. The pipeline has been conceived to allow comparative analysis of FNTDCs by standardizing, as far as possible, prerequisite databases (e.g. annotation pipeline and genome scanning parameters). This allowed to gain an overview of the impact of tandem genes on the number of “functional cluster” and to focus on desired stringency threshold when looking for FNTDCs. Furthermore, special care was given at assessing the effect of various parameters related to tandem duplicates gene call, *P-value* for hypergeometric test and scanning windows size and positioning.

Finally, rich datasets for detected cluster are reported to allow the reader to look for desired clusters and function in a frictionless way. These files include, at the various stringency settings, graphic and spreadsheets summaries of the FNTDCs patterning and FNTDCs detail regarding annotation, Blast results, excluded tandem duplicated genes and multiple alignments. Additionally, the whole set of summaries and raw files is available at https://figshare.com/s/7137f9c33c93ce7c76bd.

## Materials and methods

### Genome releases and annotations

The following genome releases and associated protein models were employed: rice (*Oryza sativa japonica*, cv. Nipponbare) IRGSP-1.0 ensembl genome release 32; arabidopisis (*Arabidopsis thaliana*, col-0) TAIR 10; grapevine (*Vitis vinifera*, IGGP 12x ensembl genome release 33). It should be noted, however, that in most cases genome-wide gene annotations rely on specific pipelines where queries are searched against databases not uniformly updated that may differ in terms of extent and curation. Furthermore, different threshold parameters for blast hits identity and number, and gene length may be employed. Thus, for reason of consistency, to reduce annotation pipeline-based biases that could hamper a solid interspecies comparison, default annotations as available in public databases were discarded, and genes were re-annotated with a unique, common and updated pipeline (Blast2GO PRO, version 4 [19]) using as query predicted proteins (including isoforms, if present) against NR *viridiplantae* database and otherwise default parameters. To retrieve all possible annotations, the proteins were blasted against the NR database (NR update: October 2016) and Interpro scans were run to gain further GO tags which were merged to the main pool of GO tags. Then, annex (augmentation of annotation tool option available in Bast2GO) was used resulting in further augmentation of annotations and obsolete GO-ids were removed. In preliminary tests with rice, our annotation pipeline retrieved 126,864 GO tags for 27,220 genes, while the default annotation available in ensembl plants databases (mart 31, 2017) provided 121,444 GO tags for 21,708 genes, indicating that few thousands of genes were lacking GO tag annotation when compared to our pipeline. In rice, all well-known (experimentally validated) FNTDCs were detected making use of our custom annotations as GO inputs for the pipeline, while the same result was not achieved when the default annotations were employed. Therefore, *a fortiori*, based on the compulsory need of a unique, consistent gene annotation pipeline, we adopted the novel pipeline for all three genomes. The annotations including GOs of the three domains (BP, CC and MF) were collected and, in case more than one isoform was present for a given gene, all retrieved GO by all isoforms were assigned to the single, representative longest isoform subsequently used for Blast tandem duplicate searches and GO hypergeometric testing. The novel annotations are available at https://figshare.com/s/7137f9c33c93ce7c76bd. In fact, tandem duplicated genes must be identified and reduced to one representative member to rule out false positive FNTDC call due to the presence of tandem duplicated genes sharing the same GO. Therefore, upon identification of tandem duplicated genes, only the first gene member of the tandem (based on ascending chromosome coordinates) should be considered for GO enrichment testing as a “placeholder” for all tandem genes and would count as only one gene in enrichment tests. Homology detection criteria were devised according to various degrees of stringency, considering that no clear-cut boundaries can be defined since genuine tandem duplicates can present large indels while, as well as few amino acid changes that pose the basis for gaining a novel function.

### Scanning windows and GO enrichment tests

**Fig 1:**
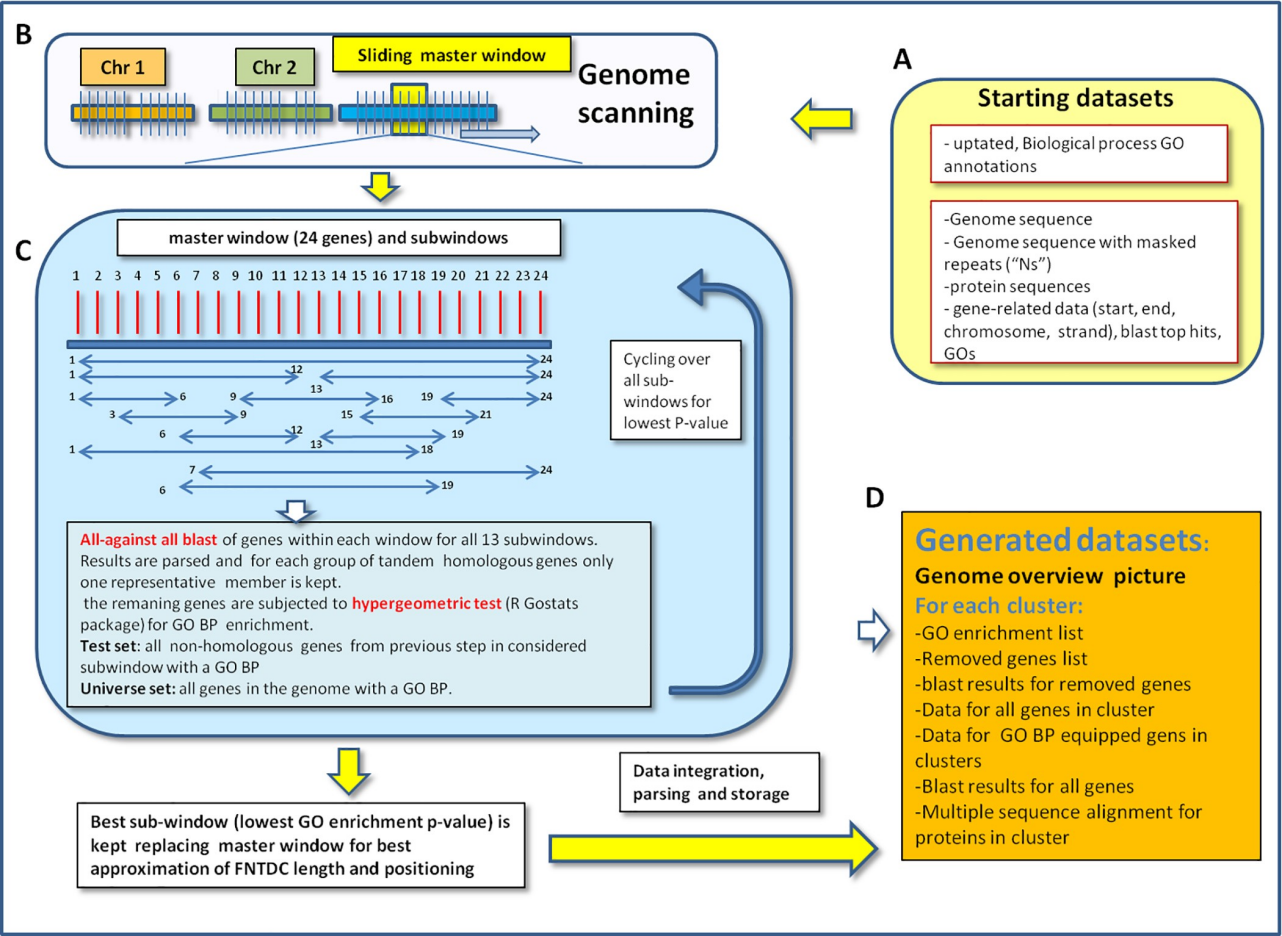
Scheme representing the FNTDC detection pipeline. **A:** prerequisite databases. B: scheme of master scanning window. **C:** details of cycling over sub-windows including homology detection, GO enrichment calls and looping to determine best (lowest *P-value*) sub-window. **D**: final output datasets.

Due to the presence of transposable elements, repeats and various forms of non-coding DNA, gene density dramatically varies among plant genomes. Therefore, the scanning window for a comparative study cannot be defined by a fixed fragment length. We therefore set, as scanning window, a defined number of genes, since coding regions are much more conserved among genomes due to selective issues (Fig 1). Starting from a sliding master windows of 24 GO-BP–assigned genes, 13 sub-windows were tested. At each subsequent step, the master window moves forward to cover the next, adjacent 24 GO-BP–assigned genes (i.e., genes 25 to 49). In Fig 1C, master window genes have been labelled from 1 to 24 and the positioning of 13 sub-windows covering from 6 to 24 genes is indicated. The master window itself is considered, in the pipeline, *de facto* as a sub-window and is compared with the remaining sub-windows for best GO enrichment. The shortest sub-window was set to six genes to reduce the number of split FNTDC and contain the overall number of sub-windows to be tested. Since 24 genes can be considered a relatively large size (currently, plant functional cluster are reported to consist from 3 to 10 genes [1]), most FNTDC are likely to be identified and fine-tuned to a more precise location and gene content by this sub-window testing procedure, resulting in lower *P-value* GO enrichment calls. Some very large FNTDCs may be split in two but they would still be identified owing to the presence of several genes with the same GO in both split clusters. With respect to cross-species comparison, the same tuneable, sub-windowing mechanism will act to minimize difference in FNDTCs positioning and offsets (when coupled with the exclusion of the major noise represented by non-coding DNA). Finally, based on such fine-tuning of window size and positioning, the sub-window (or the master windows itself) displaying, if any, the lowest *P-value* for enriched GO was then considered as the best approximation of functional cluster length and positioning associated to the parent master window region. GO enrichment was conducted via a hypergeometric test as implemented in Bioconductor package GO-stats [20] version 2.42.0 with values ranging from *P-value* ≤1e^-5^ up to *P-value* ≤1e^-8^. According to most recent and common views of a prototypical functional cluster [1 and reference therein] and in order to avoid false positives, a minimum of 3 genes sharing the same GO-BP was set as prerequisite condition to call for a candidate FNTDC. Only GO-BP (Biological Process-assigned) genes along the entire genome (universe set) as well as in the test set (genes contained in the window) were considered.

### Tandem duplicates detection

In advance of each GO enrichment test, tandem duplicated genes were detected by an all against all BLASTP (*blast2*; version 2.2.26, gapped alignment, threshold expect value = 10, otherwise default parameters). In the case of genes with more than one isoform, the longest isoform was considered as representative for the corresponding gene in blast analyses. Four different settings for tandem duplicate genes detection via blast analysis were implemented. All genes within a defined candidate window were analyzed “all against all”, using specific threshold sequence identity and identity region length to filter the results. The most stringent tandem detection criteria were implemented by *setting_1* where a minimum of 40% amino-acid identity over at least a ratio of 0.4 (both in terms alignment length to query length and alignment length to subject length) was required to call a tandem duplicate. Less stringent criteria were implemented by *setting_2* (70% identity and ratio 0.7), *setting_3* (90% identity and ratio 0.9) and *setting_4* (98% identity and ratio 0.98). When one or more tandem duplicated genes were identified, the first (based on ascending genomic coordinates) gene of the tandem was kept as representative of all tandem duplicated genes and all remaining genes above the threshold were discarded.

### Multiple sequence alignments

To visualize protein similarities, the MSA bioconductor package [21] was used to display multiple alignments for GO-BP–assigned proteins (expect value > = 10) according to clustalW algorithm highlighting similar and >50% conserved residues. Identified clusters files

For in-depth analyses, as detailed in Tables 1 and 2, for each genome and tested conditions (i.e. combinations of homology detection criteria and *P-value*s) the following data have been released through figshare at https://figshare.com/s/7137f9c33c93ce7c76bd or are available in main text as specified below.

**Table 1:** List of provided raw annotation files and summary of their content. Data available in figshare: https://figshare.com/s/7137f9c33c93ce7c76bd.

File suffix	Summary of contents	Column headers *(if applicable)*
cluster number_SUMMARY_OVER	Output results for GO hypergeometric enrichment test (R Bioconductor GOstats package)	GOBPID
		P-value
		OddsRatio
		ExpCount
		Count
		Size
		Term
cluster number–GO descriptions–DATA_GENES_WITH_BP	ID chromosome, position, strand and B2GO derives data (descriptions (including B2GO topblast results, and GOs) for GO-BP equipped genes within cluster	Gene.stable.ID
		Sequence_desc.
		TERMS
		Chromosome.scaffold.name
		Gene.start..bp.
		Gene.end..bp.
		Strand
		Sequence_length Hit_desc.
		Hit_ACC
		E-Value
		Similarity
cluster number–GO descriptions–DATA_ALLGENES	ID chromosome, position, strand and B2GO-derived data (descriptions (including B2GO top-blast results, and GOs) for all genes and GO 4domains (if available) in cluster	Bit-Score
		Alignment_length
		Positives
cluster number–REMOVED_BLAST	Reports, if present, ID(s) of genes removed due to homology criteria prior of hypergeometric testing for GO enrichment call	ID of gene(s) or NONE
cluster number–BLAST____FILTR_	Reports blast output for genes filtered out based on chosen sequence homology parameters	Query_id
		len_query
		Subject_id
		len_subject
		identity alignment_length
		mismatches
		gap_openings
		q_start
		q_end
		s_start
		s_end
		e-value
		bit_score
		len_algn_VS_len_query
cluster number–BLAST____EVERYTHING	Reports blast output for all tested genes (blast outputs showing expect value < 10)	len_algn_VS_len_subj
		len_query_vs_len_subj
		len_algn_minus_gaps_vs_len_subj
		len_algn_minus_gaps_vs_len_query
		Query_id
cluster number–ALIGNMENT.pdf	Multiple sequence alignment (clustalW algorithm) of protein genes in cluster with a GO BP tag. Similar and >50% conserved residues are highlighted	-
Genome snapshot files.png	Within each main condition folder, six snapshots (png files) at different sizes and resolution showing a qualitative snapshot of FNTDC patterning, as also related to genome repeats. Selected snapshots at intermediate settings (setting_2) are also directly visible and available in figshare main page link	-

**Table 2:** List of provided summary files and description of their content. Data available in figshare: https://figshare.com/s/7137f9c33c93ce7c76bd.

File type	Summary of contents
Single summary files	For each condition and genome, the files summarise in a single worksheet all main data gathered from the 6 files of each FNTDC in raw data
Merged summary files	For specific subset of genomes and/or conditions, the files summarize in as many worksheets as necessary all main data gathered from the 6 files of each FNTDC in raw data e.g. all genomes and all conditions. Only one genome with all conditions; all genomes and only stringent conditions; etc.
Common and specific GOs files	The file details which GO BP tags are common vs specific for one genomes or combinations of genomes at *P-value* 1e^-6^ and 40, 70, 90 and 98% stringencies. Previously published data from *T*. *durum* Svevo are also included for comparative purposes.

## Results and discussion

Despite the strong interest in secondary compounds clusters, which has prompted the recent development of several dedicated tools, much less information on functional cluster covering all biological functions, including primary metabolism compounds, is available. Such and *ab-initio*, GO-BP based systematic genome scanning is valuable for assessing the entirety of functional gene clustering and therefore genome plasticity mechanisms and neofunctionalization phenomena. Furthermore, given the persistent lack of functional information in more than one-third of genes in plant genomes, entanglement of a yet unknown gene in a FNTDC can provide hints toward it function. Finally, the database parts produced at relaxed settings for tandem duplicated gene exclusion are a valuable resource for search and identification of gene clusters based on a desired function, irrespective of the presence or not of tandem duplicated genes.

### Parameter testing and optimization for FNTDCs detection

#### Annotation quality

Since the functional classification of genes is based on a dedicated annotation, the availability of a reliable, fine-grained and updated annotation is mandatory. Furthermore, to allow conserved interspecies cluster identification, defining a reproducible standard for annotation is highly desirable. The annotations available in the genome data repositories are often inconsistent in terms of depths and confidence and do not allow a clear comparison among different genomes. In fact, different criteria, databases, and personal curation were adopted from case to case. We, therefore, resorted to a unique, state-of-the-art tool for performing standardized and updated re-annotations, namely Blast2GOpro [19] which also allowed to select the appropriate subsets of reference databases (*viridiplantae*) to minimize annotation artefacts. Re-annotation of arabidopsis (TAIR 10), rice (IRGSP-1.0), and grapevine (IGGP 12x) genomes was carried out as indicated in Construction and Content section leading to several improvements. E.g., in the rice genome we could identify the well-known diterpene clusters (momilactone and phytocassane clusters [22, 23] which were not tracked when the pipeline was fed with official (plant ensembl biomart 31) annotations. The whole FNTDC detection pipeline is presented in Fig 1.

#### Scanning master window optimization

Frequently, genome scanning approaches consist in defining a window size (e.g. fixed DNA interval in kb) and a sliding offset with or without some overlap between adjacent window positioning. However, since this work aims at the identification of a cluster of genes, a window made of a fixed number of genes, rather than a fixed kb size, was employed. Furthermore, as very few or even no genes are present in large genomic regions associated with repeats (TE-associated, microsatellites, heterochromatic areas), and since the repeat content vary widely across the genomes, a cross species comparison is only possible when FNTDCs are searched with windows based on a fixed gene number. After some preliminary tests, a scanning master window of 24 GO-BP assigned genes (tested values for master window ranging from 12 to 40 genes) was set as it yielded the highest number of non-duplicated FNTDCs. Considering that most plant FNTDC clusters identified to date range between 3 and 10 genes [1], too short master windows could result in splitting some FNTDCs, while too large master windows (e.g. 40 genes) could result in some false negatives. Large genomic regions are more prone to harbour more than one cluster, although only one FNTDC for master window (the one with associated lowest *P-value* for GO enrichment) can be identified by our pipeline. The sub-window fine tuning was found beneficial to rule out false negative FNTDCs. In fact, misplaced sub-windows, or sub-windows missing some components of the putative FNTDC or sub-windows covering too many genes with respect to the actual set of genes being effectively part of a FNTDC, could result in false negative GO enrichment calls and/or incorrect GO enrichment *P-value* and, ultimately, in a failure in the detection of FNTDCs.

#### Tandem duplicated genes identification criteria

Prior to each GO enrichment testing, tandem duplicated genes were detected by all against all BlastP analysis. The first occurring gene member (based on ascending genome coordinates) of each tandem duplicate gene pool was kept as representative of all cognate tandem duplicated genes, while the remaining duplicated genes were discarded from GO enrichment tests to avoid false positive FNTDC calls. Four different settings were tested to call for tandem duplicates. *Setting_1*: 40% identity over at least a ratio of 0.4 (both alignment length to query length and alignment length to subject length); *setting_2*: 70% identity and ratio 0.7; *setting_3*: 90% identity and ratio 0.9; *setting_4*: 98% identity and ratio 0.98. The settings for homology detection were conceived to test a wide range of conditions. The stringent settings by defining an as low as 40% identity threshold over 0.4 of sequence length ratio, scores as duplicate also hidden tandem duplicate genes. This happens when, due to the time elapsed from the duplication event, the genes accumulate mutations, deletions and insertions leading to some substantial divergence in their sequence without a real functional diversification. These genes, despite apparent sequence dissimilarities, still maintain a tandem duplicate nature and share the same GO-id and function and must be regarded as tandem duplicates. Stringent settings, therefore assume and detect as tandem duplicates, also genes with just moderate sequence similarity, leading to a large fraction of detected tandem duplicates. As these tandem duplicates would produce false positive FNTDC calls, they are filtered out. Overall, this result in fewer call of FNTDCs. Such settings are expected to provide high rates of true positive FNTDCs while leading to a significant number of false negatives. On the opposite, more tolerant settings (e.g. *setting_3* and *setting_4*), identify as tandem duplicates only genes exhibiting high similarity (90% and 98% identity and 0.9 and 0.98 of sequence length ratio, respectively). Tolerant settings lead to fewer genes detected as tandem duplicates and, in turn, more FNTDCs, indeed, under these settings, tandem duplicated genes retained in the analysis easily fulfil the prerequisite threshold of at least 3 co-localized genes sharing the same GO-BP. Consequently, *setting_4*, by preserving most tandem duplicated genes, leads to a large reference superset of all co-localized genes sharing a GO-BP (including cluster consisting solely of tandem duplicates). Noteworthy, tolerant settings allow the investigation of FNTDCs made of genes with barely detectable differences where neo-functionalized genes result from few amino-acid substitution(s) [24, 25].

In general, we refer at *setting_2* (70% identity, ratio 0.7, *P-value* of 1e^-6^) as the “intermediate” settings and, for all settings, unless otherwise stated, we refer to the *P-value* of 1e^-6^. Genome snapshot files providing a qualitative overview of FNTDCs crowding over genomes are available for rice, arabidopsis and grapevine, at various details and magnification as indicated in Table 2. These snapshots depict the FNTDCs labelled for reference to corresponding spreadsheet and alignment FNTDC files but mainly allow to estimate FNTDCs positioning with respect to interspersed repeats and low complexity regions (as detected with the RepeatMasker tool) which are marked in green below chromosomes.

### Pipeline validation

The pipeline developed in this work differs from the recent secondary-metabolism targeted procedures based on libraries of pHMMs [5, 7, 8]. Making use of gene GO-BP tags as input data, our pipeline allows to target also primary metabolism, although it is less effective in tracking secondary metabolism enzymes. Besides the input data, the two procedures also differ in several parameters such as the minimum number of genes, homology tolerance to tandem duplicates and scanning mechanisms.

Given that *in silico* procedures for FNTDC detection can only provide best candidate clusters awaiting for experimental validation, a true validation of the pipeline can only be achieved through a comparison with experimentally proved functional clusters. Furthermore, in experimentally validated FNTDCs the proven function of cluster member genes has in most cases allowed annotation and propagation over public databases of gene function and GO-BP tags (i.e. the input data for our pipeline) for the same genes as well as homologous genes. Therefore, we considered manually curated and experimentally validated FNTDCs as the most reliable validation set for the new FNTDC detection pipeline. Among 14 well-characterized plant FNTDCs [3, 4], two were reported in rice and two in arabidopsis (none for grapevine). In rice, phytocassanes and momilactone terpenoid clusters, localized in chromosome 2 and 4, respectively, [22, 23] were described in the context of studies on rice response to the fungal pathogen *Magnaporte oryzae*. Both clusters were called by our pipeline even at most adverse *P-value* and stringency setting (i.e. *setting_1*, *P-value* 1e^-8^ id: FNTDC#2 and FNTDC#3, respectively). At intermediate setting (*setting_2*, *P-value* 1e^-6^) the ids are FNTDC#26, OS02G0568700_TO_OS02G0572050 on chromosome 2 for phytocassanes and FNTDC#45, OS04G0178300_TO_OS04G0181300 on chromosome 4 for momilactones. Noteworthy, in preliminary attempts making use of the default mart 31 (2017) GO annotation for rice (as retrieved by public databases), we could not identify the diterpene clusters. In Arabidopsis, thalianol triterpene cluster on chromosome 5 [26] was correctly identified by our pipeline (*setting_2*: FNTDC#61, AT5G47930_TO_AT5G48020). On the other hand, it was not possible to identify the triterpene marneral cluster [27], since both official TAIR 10 and our Blast2GOpro-based annotations failed to associate the terpene-associated GO tags to the relevant CYP genes (e.g. AT5G42590, AT5G42580). We conclude that our pipeline identified all the three long-known functional clusters which, based on input data GO-tags availability, had the potential to be identified. On the other hand, with respect to reliability of our pipeline in cross-species genome analyses, in our previous report in durum wheat, when faced to the anciently duplicated A and B genomes, we could identify, over a total 197 candidate FNTDCs, 84 FNTDCs displaying a mirror, homeologous counterpart in the two genomes [6], indicating that our pipeline was effective in identifying expected similarities in FNTDC patterning over different sub-genomes.

### Impact of homologous gene exclusion over FNTDC calls

**Fig 2:**
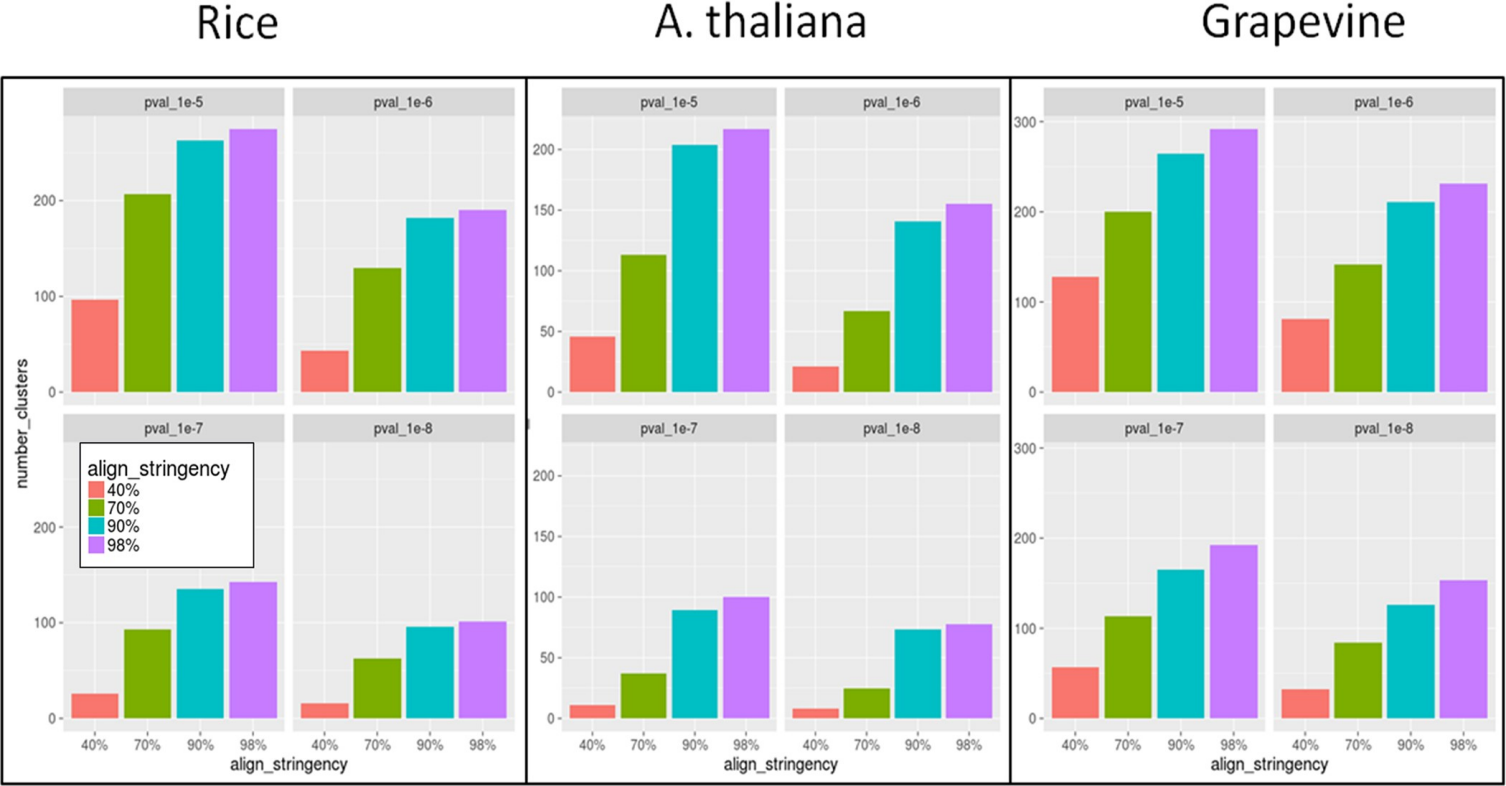
Effect of homology detection stringency (tandem genes exclusion) and *P-value* threshold on the number of called FNTDCs. Alignment stringency of 40% refers to a minimum 40% identity and alignment length to query and alignment length to subject ratio of at least 0.4. *P-values* are minimum *P-value* thresholds for GO-enrichment hypergeometric test.

For all tested genomes, homology detection criteria had a strong impact over the number of called FNTDCs (Fig 2). This is not surprising, as genomes are well known to be rich in tandem duplicated genes due to genome rearrangements such as segmental duplications [28]. Tandem duplicated genes are, by themselves, an interesting topic to explore and dedicated databases have been created (PTGBase [29]). By adopting virtually no filtering over sequence heterogeneity (i.e. *setting_4*) in rice genome very little genes were discarded (only 3% of identified cluster had at least one discarded gene prior to hypergeometric GO enrichment test) and 275 FNTDC were called at a *P-value* of 1e^-5^. On the other hand, *setting_1* combined with the same *P-value* of 1e^-5^, resulted in only 97 FNTDCs. *Setting_1* and *setting_4* define the range boundaries of possible FNTDCs, while *setting_3* (90% identity and ratio 0.9 thresholds) is probably still prone to some false positives with some 182 FNTDC (*P-value* 1e^-6^). We point to *setting_2* as the most reliable estimation of effective FNTDCs with 207, 130, 93 and 63 clusters called at *P-value*s of 1e^-5^, 1e^-6^, 1e^-7^ and 1e^-8^, respectively. We refer to 70% homology (*setting_2*) and *P-value* of 1e^-6^ as an intermediate reference value. When the same approach was applied in Arabidopsis 113, 67, 37 and 25 FNTDCs were called at *P-value*s of 1e^-5^, 1e^-6^, 1e^-7^ and 1e^-8^, respectively. Finally, in grapevine, 200, 142, 113, 84 FNTDCs were called at *P-value*s of 1e^-5^, 1e^-6^, 1e^-7^ and 1e^-8^ (*setting_2*). Details for all settings are reported extensively in summary tables and raw data files (see Tables 1 and 2 for file types)

### Cluster sizes

**Fig 3:**
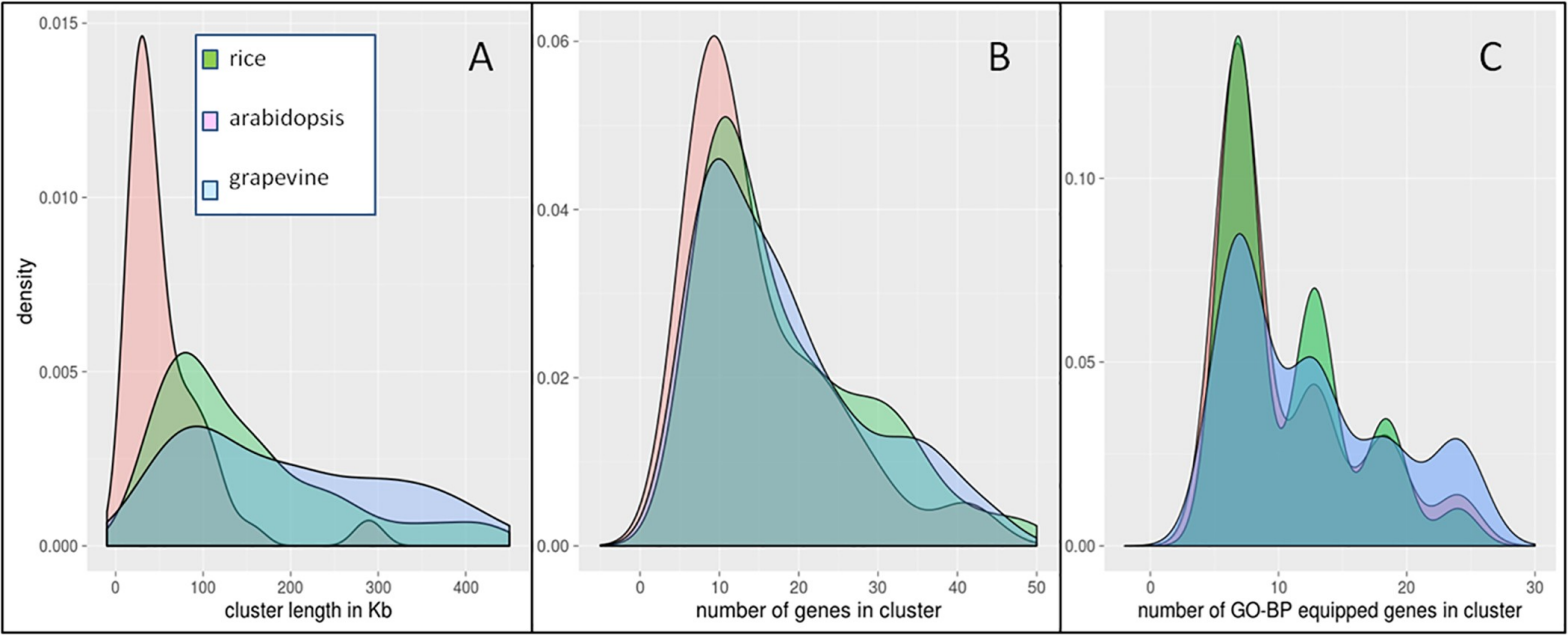
Cluster size estimates. Length distribution of FNTDCs measured according to various parameters. **A:** length of FNTDCs as measured in spanned Kb. **B:** sum of all genes within FNTDCs, including unknown genes. **C:** sum of all genes within FNTDCs equipped with a GO biological process. Tandem duplicates are not excluded from sums.

The distribution of FNTDC sizes as measured from different parameters (length in spanned kb, number of genes, number genes in FNTDCs equipped with a GO BP) substantially appears to be proportional to genome sizes (Fig 3). Arabidopsis shows a major peak around 25 kb, while rice and grapevine around 80–90 kb despite showing less sharp peaks, especially grapevine. These lengths appear consistent with known higher proportions of non-coding DNA in intergenic regions for the larger genomes, as also supported by the 900 kb average length previously detected for durum wheat [6]. Noteworthy, when only GO-BP equipped genes are considered, the discontinuous nature of sub-windows gene sizes emerges with discrete peaks (Fig 1C). As tested in early trials with small genome chunks, this patterning disappears when all possible sub-windows are probed. However, this approach turned out to be very computationally demanding, as it required to test 253 sub-windows for each master window (considering all possible sub-windows sizes, from 3 to 24) and positioning.

### Major identified FNTDCs

While it would not be feasible to analyse in detail all FNTDCs, upon manual scrutiny consisting in verification of reliability of assigned gene functions, gene positioning and annotations, we report hereafter several arbitrary but significant examples of *bona fide* FNTDCs. In fact, in some cases, the combination of co-localization and strong divergence in sequence may lead in our database to potential false FNTDC calls due to, e.g., large protein families of evident tandem origin, whose members may have diverged in sequence substantially to escape filtering for sequence similarity (e.g. cluster #57, the highly polymorphic self-compatibility loci “recognition of pollen”; [30]). All the following called FNTDCs can be tracked and analyzed in detail using the summary files and the raw files available at figshare (https://figshare.com/s/7137f9c33c93ce7c76bd), where #id number corresponds to the one identified at intermediate settings (70% homology and *P-value* of 1e^-6)^.

#### Rice example 1: Proteasome-related clusters

Three distinct FNTDCs with top enriched GO: SCF-dependent proteasomal ubiquitin-dependent protein catabolic process (GO:0031146) have been detected on chromosome 4, 9 and 10. In *setting_2*, these correspond to FNTDC#46 (OS04G0190000 to OS04G0194400), FNTDC#99 (OS09G0341500 to OS09G0344500) and FNTDC#107 (OS10G0137700 to OS10G0147900). FNTDC#107, after homologous gene discarding, contains nine of the 111 rice genes exhibiting the GO term”SCF-dependent proteasomal ubiquitin-dependent protein catabolic process (GO:0031146)”. Furthermore, the cluster includes a protein (OS10G0141400) with as Blast best hit: “26S proteasome non-ATPase regulatory subunit 4 [*Zea mays*]” and with associated GOs: “proteasome-mediated ubiquitin-dependent protein catabolic process—proteasome assembly” as well as other proteins with top hits to: “F-box domain containing expressed”and with associated GOs: “protein ubiquitination SCF-dependent proteasomal ubiquitin-dependent protein catabolic process”. FNTDC#99 on chromosome 9 (OS09G0341500 to OS09G0344500) carries four genes exhibiting the GO term”SCF-dependent proteasomal ubiquitin-dependent protein catabolic process (GO:0031146)”, all of them with a GO cellular component tag “plastid”. This may be of interest in view of the findings about chloroplast-localized E3 ligase and on the more general control of chloroplast biogenesis by proteasome system [31, 32].

#### Rice example 2: Mismatch repair cluster

FNTDC#59 is localized in chromosome 4 and is enriched in “mismatch repair” GO (GO:0006298). The cluster (OS04G0680000 to OS04G0682900) contains 4 of the 19 rice genes tagged with “mismatch repair” GO BP. The genes display the following Blast best hit tags: RNA polymerase II transcription factor B subunit 2; probable allantoinase; Os04g0680700 [*Oryza sativa subsp*. *japonica*], (i.e. hits to himself, but very recent searches annotate it as DNA mismatch repair protein MutS); DNA mismatch repair MSH3.

#### Rice example 3: TOR signalling clusters

FNTDC#115 and #126 both contain 3 genes each of the 7 rice genes tagged with “TOR signalling” GO BP. Consistent with known duplications in rice genome [33] the two clusters appear to closely mirror each other and are localized, respectively, on chromosome 11 (OS11G0109301 to OS11G0109950) and 12 (OS12G0109200 to OS12G0110100). In both FNTDCs, two genes display the following Blast best hit tag: “regulatory-associated of TOR 2” while the third gene is annotated as “Double-stranded RNA binding motif family expressed [*Oryza sativa subsp*. *japonica*]. Multiple alignments reveal very poor sequence similarities ruling out recent tandem duplicated origin and supporting divergences, possibly to acquire neo-functionalization.

#### Arabidopsis example 1: Pseudouridine synthesis cluster

FNTDC#07 located in chromosome 1 from AT1G20280 to AT1G20470, contains 4 of the 30 arabidopsis genes tagged with GO BP “pseudouridine synthesis”. This GO refers to the intramolecular conversion of uridine to pseudouridine in and is a post-transcriptional base modification occurring in tRNA, rRNA, and snRNAs. The four genes exhibiting this GO tag are annotated as follows: F-box kelch-repeat At1g20940; mitochondrial import inner membrane translocase subunit TIM17-1-like; tRNA pseudouridine synthase mitochondrial-like.

#### Arabidopsis example 2: Positive regulation of translational elongation cluster

FNTDC#08 contains 3 of the 6 arabidopsis genes tagged with GO “positive_regulation of translational elongation” (chromosome 1, AT1G26610 to AT1G26680). Further, enriched GO BP tags for the same FNTDC are (in decreasing order of *P-value*): positive regulation of translational termination, translational frameshifting, positive regulation of protein complex disassembly. Proteins within this FNTDC with the GO BP translation-related as listed above have as best BlastP hits proteins annotated as: zinc finger ZAT4-like [*Brassica oleracea oleracea*]; eukaryotic translation initiation factor 5A-2 [*Camelina sativa*]; UXT homolog; Mediator subunit Med10 [*Arabidopsis thaliana*]; B3 domain-containing REM17-like isoform X1. In addition to the above diverging annotations, the alignment files further display strong sequence heterogeneity.

#### Arabidopsis example 3: Microtubule nucleation cluster

FNTDC#19 contains 3 of the 23 arabidopsis genes genome tagged with GO “microtubule_nucleation” (chromosome 1, AT1G80245 to AT1G80320) with further associated GO BP tags as: microtubule polymerization; microtubule polymerization or depolymerisation. The proteins within this FNTDC sharing the above GO BP exhibit as best Blast hits Spc97 Spc98 family of spindle pole body (SBP) component [*Arabidopsis thaliana*]; gamma-tubulin complex component 5-like; pentatricopeptide repeat-containing [*Arabidopsis thaliana*].

#### Arabidopsis example 4: DNA topological change cluster

FNTDC#053 contains 3 of the 22 arabidopsis genes tagged with GO “DNA_topological_change” (chromosome 5, AT5G04130 to AT5G04190). The corresponding proteins exhibit as best Blast hits DNA gyrase subunit mitochondrial; transcription factor bHLH101 [*Arabidopsis thaliana*]; alpha carbonic anhydrase 3-like. Protein sequences substantially differ as shown in corresponding multiple alignment file.

#### Grapevine example 1: Actin filament bundle assembly

FNTDC#39 contains 3 of the 6 genes in the grape genome tagged with top GO “actin filament bundle assembly” (chromosome 06, VIT_06s0004g06430 to VIT_06s0004g06520). The best hits for the three relevant genes with the associated GO tags are: villin-1-like [*Gossypium hirsutum*]; non-specific phospholipase C1; ammonium transporter 1 member 3-like. Blast results and the multiple alignment reveals strongly heterogeneous sequences pointing to non-tandem or at least dramatically diverging structures.

#### Grapevineexample2: Thiamine diphosphate metabolic process

FNTDC#45 contains 3 of the 4 genes in the grape genome tagged with top GO “thiamine diphosphate metabolic process (chromosome 07, VIT_07s0005g00190 2 to VIT_07s0005g00270). The best hits for the three relevant genes exhibiting the above GO tag are: thiamine pyrophosphokinase 1 isoform X1 [*Ziziphus jujuba*]; DNA mismatch repair PMS1; Small nuclear ribonucleo family isoform 1 [*Theobroma cacao*]. Again, blast results and the multiple alignment reveals strongly heterogeneous sequences pointing to non-tandem or at least dramatically diverging structures.

#### Grapevine example 3: Mismatch repair

FNTDC#99 (followed by further enriched GOs: cellular response to stress; DNA repair; cellular response to DNA damage stimulus) contains 4 of the 19 genes in the grape genome tagged with top GO “mismatch repair” (chromosome 14, VIT_14s0006g00910 to VIT_14s0006g01030). The best hits for the four relevant genes with the associated GO tags are: DNA repair complementing XP-C cells homolog isoform X2; SPIRRIG-like isoform X2; DNA mismatch repair MLH1 isoform X2; DNA repair complementing XP-C cells homolog.

### Unknown genes interspersed in FNTDC

Many Arabidopsis genes, one of the most studied reference species, are still of unknown or uncertain function ([17, 18]). This indicates that still a substantial fraction of genes escapes our functional understanding and any hints pointing to a candidate annotation for this large group of genes should be valuable. As for the case of the maize gene *BX8*, located in close proximity of maize DIMBOA FNTDC [34] with unknown function until [35], functionally unknown genes embedded in a cluster may reveal, upon subsequent investigations, to be active part of the cluster and therefore share it functions.

By design, our gene sub-windows represent predetermined discrete values (thirteen sub-windows of length from 6 to 24, as detailed in Fig 1C, differentially positioned within the boundaries of a master window of 24 genes). Based on the GO enrichment Hypergeometric test output, the sub-window producing (if any) the lowest *P-value* for GO enrichment is selected and thus a fine-tuning (over the master window) for gene number and positioning is performed. Therefore, the genes within the selected, refined sub-window (including interspersed or adjacent unknown genes) appear as good candidates for sharing the same function(s) as the genes of known function within same sub-window.

**Fig 4:**
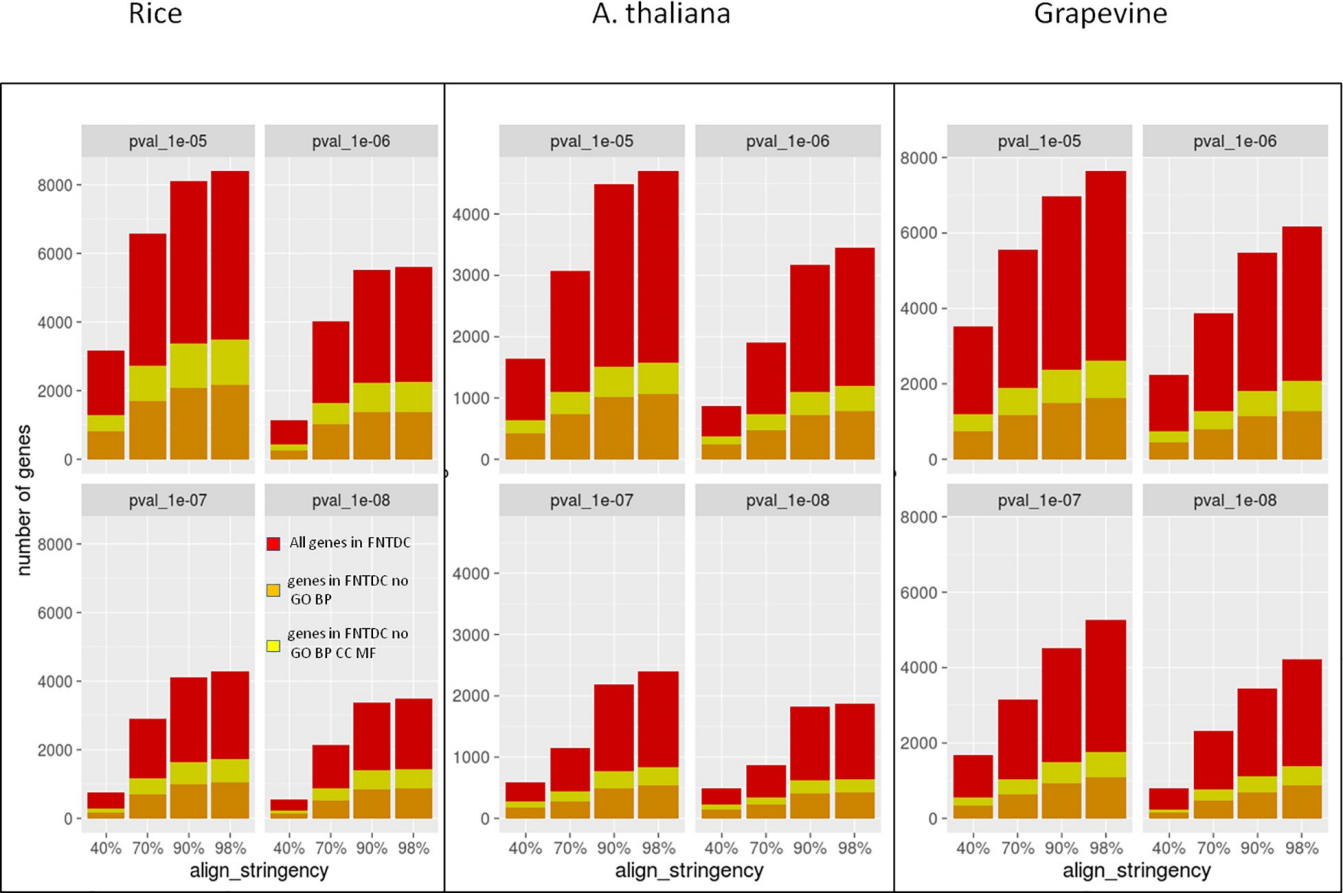
Unknown genes embedded in FNTDCs in rice, arabidopsis and grapevine. The stacked barplots display the total number of genes (red bars), the subset of genes devoid of GO Biological Process (NO_GO_BP; orange bars) and the subset of genes devoid of any GO tag for all ontology domains (NO_GO_BP_MF_CC, yellow bars). Only genes embedded within called FNTDCs at the specified homology detection stringency (align_stringency; x axis) and *P-values* as specified in sub-plot titles are considered. Cumulative values are shown (genes in subsets are not subtracted from supersets).

Intriguingly, in rice, at the intermediate settings (*setting_2*, 70% *P-value* 1e^-6^), over the total of 2,409 genes included in the FNTDCs (on average 18.53 genes/cluster), 1,014, (7.8 genes/cluster) are devoid of GO BP annotation, implying no functional understanding of the gene, and 612 (4.7 genes/cluster) are devoid of any GO domain tag as GO BP, MF and CC, indicating lack of any kind of information. Similarly, in Arabidopsis, we identified 1,177 genes participating to FNTDCs (17.56 genes/cluster) and among them 472 were devoid of GO BP annotation (7.04 genes/cluster) and 259 of any GO domain tag (3.86 genes/cluster). Finally, in grapevine, out of 2,594 genes involved in FNTDCs (18.26 genes/cluster), 805 were devoid of GO BP annotation and 485 of any GO domain tag (5.66 and 3.41 gene/cluster, respectively) (see also Fig 4). With respect to GO-tagged genes within FNTDCs, the average number of genes sharing the GO BP associated to the called FNTDC in rice (130 FNTDCs) is 5.56 (intermediate settings), while in Arabidopsis (67 FNTDCs) is 5.01 and in grapevine (146 FNTDCs) 6.14. Further details about gene and GO annotation for each gene included in FNTDC are reported in raw cluster files with suffix “DATA_GENES_WITH_BP.tsv”, “DATA_ALLGENES.tsv” and “SUMM_OVER.tsv”.

### Common clusters among species

**Fig 5:**
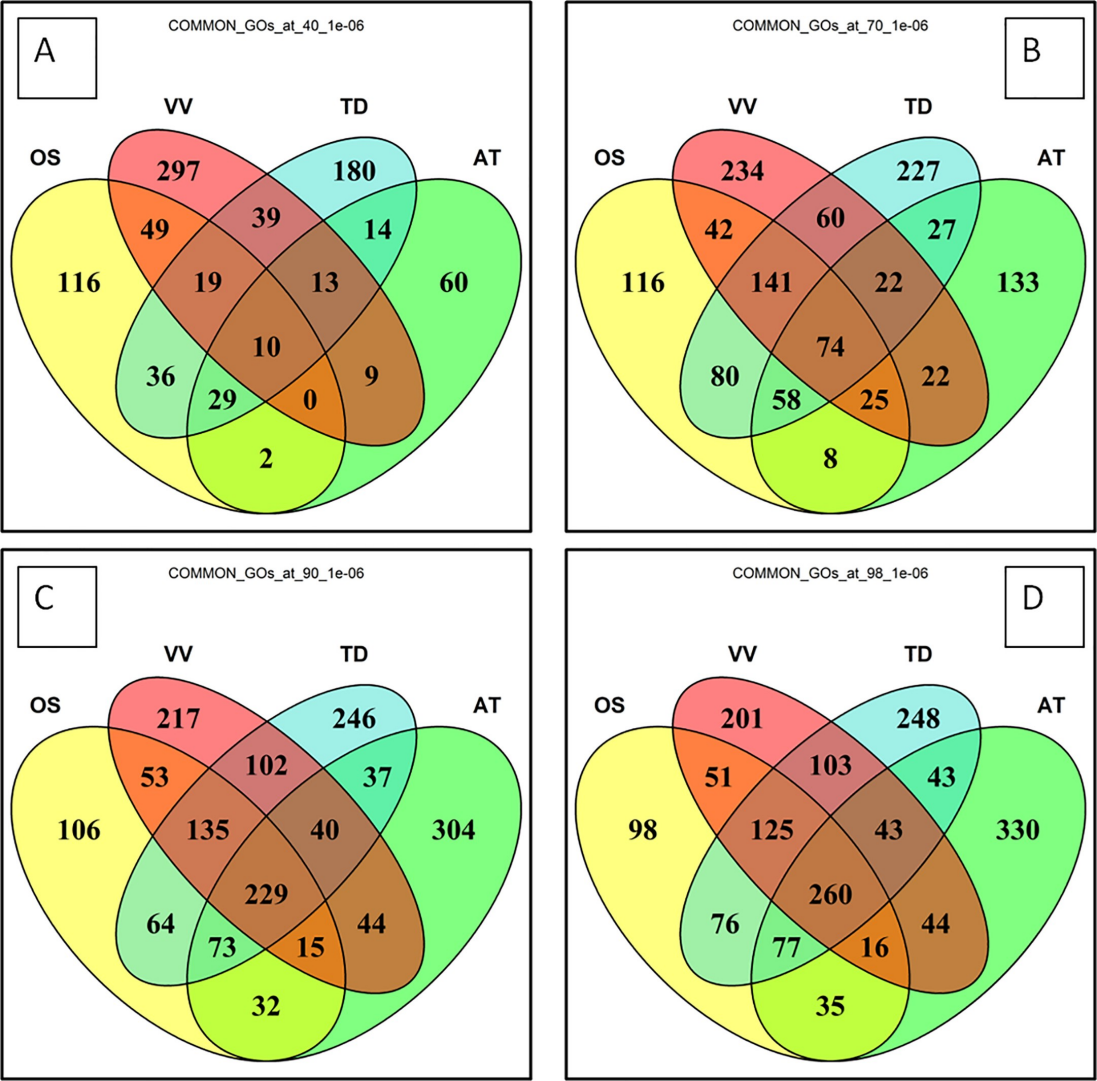
Shared GO terms. GO-BP tags associated to FNTDCs called at various identity threshold conditions and *P-value* for GO enrichment call 1e^-6^ as shared in rice, arabidopsis grapevine and durum wheat genomes. Overall GO terms number is substantially higher with respect to FNTDCs as many GO terms can be associated to a single called FNTDC. **A:** identity threshold 40% and *P-value* 1e^-6^; **B:** identity threshold 70% and *P-value* 1e^-6^; **C:** identity threshold 90% and *P-value* 1e^-6^; **D:** identity threshold 98% and *P-value* 1e^-6^.

Frequently, each FNTDC is associated to more than one GO. This because GO annotations provide fine-grained information about parent/child GOs with similar attributes as well as because of close/nested FNTDCs. Thus, we did not compress the various GO tags to just a one reference in order not to lose meaningful GO tags data. For instance, in rice, the 130 FNTDCs identified in intermediate settings (annotated after the corresponding 130 top-enriched GOs) are associated to 586 GO terms (on average, 5 enriched GO terms are associated to each called FNTDC). In most cases, such tags are closely related. E.g. in case of “xanthine metabolic process” enriched GO, also “xanthine catabolic process”, is frequently enriched, and the second is an obvious subset of the first. However, in other instances, e.g. in case of nested and / or overlapping FNTDCs, these GO tags may be unrelated. Despite these constraints and confounding elements, Venn diagrams and set operations were performed also including the data of durum wheat [6] to get a glimpse over common/private terms associated to FNTDCs (Fig 5). As expected, the most shared (353 out of 586; 61%) terms were between the two monocots, while, 99 out of 586 (16%) GO tags were shared among rice, arabidopsis and grape. A full list of common and private terms associated to the FNTDCs can be found common_and_specific_GOs file (https://figshare.com/s/7137f9c33c93ce7c76bd, see also Table 2).

## Conclusions

The dataset developed along with this work provides a rich framework for pinpointing candidate FNTDCs reflecting all GO-BP tags covering both primary and secondary metabolism. These datasets would represent of superset of functional candidate clusters, not being limited to secondary compounds biosynthetic cluster and including evolving FNTDCs. Upon further validation, FNTDCs would be of interest as starting point to elucidate genome plasticity mechanisms driving the evolution and co-localization of genes with similar function. At the most relaxed settings (98% identity), the dataset represents a large reference superset of all co-localized genes sharing a GO-BP (including clusters consisting solely of tandem duplicates) where look for specific functional regions that, based on duplications, insertion, deletions, and rearrangements, may act as neo-functionalization hotspots.

Overall, large macromolecular complexes/metabolons [36, and references therein] e.g. electron and proton transport, mismatch repair, transcription, TOR and proteasome complexes, are the most represented *bona fide* FNTDCs (i.e. not of tandem-duplicate origin) in our dataset. Noteworthy, the reactions carried by these complexes would greatly benefit from co-localization of protein components for reasons of coinheritance, local increase in concentrations of both enzyme and substrate and reduced leakage of potentially toxic intermediates. Furthermore, as found in the durum wheat genome [6], several FNTDCs are tagged with GOs referring to organelle-targeted multi-enzyme complex for proton and respiratory electron transport chain, a finding that suggest the migration of endosymbiont gene chunks towards nuclei [37] as the origins of these class of candidate FNTDCs. With respect to the timing of FNTDCs appearance, an ancient origin is likely based on the fact that a major fraction of FNTDCs appear to have formed prior of well-known duplication/polyploidization events (e.g. chromosome 11 and 12 in rice [33] and duplications in durum wheat chromosome sets A and B [6]).

## List of abbreviations

FNTDC: functional non-tandem duplicated cluster
BGC: biosynthetic gene clusters
GO-BP: Gene ontology biological process
